# Effect of Three Technical Arms Swings on The Elevation of the Center of Mass During a Standing Back Somersault

**DOI:** 10.2478/hukin-2014-0005

**Published:** 2014-04-09

**Authors:** Bessem Mkaouer, Monèm Jemni, Samiha Amara, Helmi Chaabène, Johnny Padulo, Zouhair Tabka

**Affiliations:** 1 Higher Institute of Sport and Physical Education of Ksar Saïd, Univercity of Manouba, Tunisia.; 2 School of Science, University of Greenwich, London, United Kingdom.; 3 Faculty of Medicine and Surgery, University of “Tor Vergata” Rome, Italy.; 4 Faculty of Medicine of Sousse, University of Sousse, Tunisia.

**Keywords:** Gymnastics, motion analysis, kinematics, backswing, performance analysis

## Abstract

Arms swing during standing back somersaults relates to three different “gymnastics schools”, each is considered “optimal” by its adepts. In the three cases, technical performance, elevation and safety differ. Therefore, the aim of this study was to compare the mechanical variables of three different arms swing techniques in the performance of a standing back tucked somersault. Five high-level male gymnasts (age: 23.17±1.61 yrs; body height: 1.65±0.05 m; body mass: 56.80±7.66 kg) randomly performed standing somersaults under three conditions, each following a different arms’ swing technical angle (270°, 180° and 90°). A force plate synchronized with a three dimensional movement analysis system was used to collect kinetic and kinematic data. Significant differences were observed between somersaults’ performance. The back somersault performed with 270° arms swing showed the best vertical displacement (up to 13.73%), while the back somersaults performed with 180° arms swing showed a decrease in power (up to 22.20%). The back somersault with 90° arms swing showed the highest force (up to 19.46%). Considering that the higher elevation of the centre of mass during the flight phase would allow best performance and lower the risk of falls, this study demonstrated that optimal arms’ swing technique prior to back tucked somersault was 270°.

## Introduction

It is well documented by mechanical laws that arms’ swing considerably influences performance during static and dynamic movements, particularly during jumping related exercises (Cheng and Hubbard, 2008; Hara et al., 2008a; Heinen et al., 2012). The height of the jump and its mechanical efficiency are considerably affected by the starting position at the take off, the direction of the swing and the final position of arms during the jump (Domire and Challis, 2010; Hara et al., 2008b; Marina et al., 2012). Arms swing is even more important in gymnastics’ standing back somersaults as it allows considerable height guaranteeing full 360° free rotation before safe landing (Cheng and Hubbard, 2008). Performing standing somersault is crucial in gymnastics. It is a basic skill that could be performed from standing still at the floor and balance beam routines. It could also be performed at the end of acrobatic series and/or as a dismount form several apparatus, combined with or without twists and in different body shapes (tucked, picked and straight). Performing standing back somersault relates to three different “gymnastics schools”: Russian, Chinese and Romanian, each considered “optimal” by its adepts, that affect technical performance and safety. What is the difference between them? What is the key safety factor in each one? What are the mechanical variables that influence performance in each one? This investigation is ultimately aiming to highlight these differences and clearly set the “optimal variables for safe practice and performance”.

**Figure 1.**
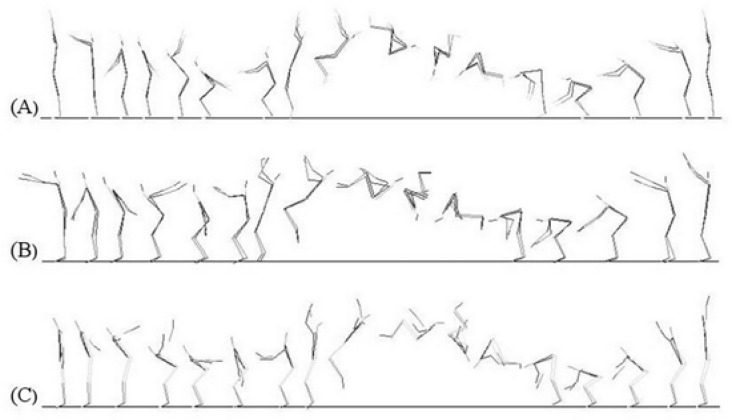
Techniques of backswing during the back somersault from a standing position. A: Backswing with 270° of arm swing (SB_s270_); B: Backswing with 180° of arm swing (SB_s180_); C: Backswing with 90° of arm swing (SB_s90_).

It is the position of the arms at backswing which is very different between the three techniques in the preparatory phase “arms swing in downward phase in”. In the Russian school, the gymnast takes off with the arms vertically pointing upwards following an oscillation (or swing) of 270° (SB_s270_) (Figure 1A). In the Chinese school, the gymnast takes off with the arms horizontally pointing forwards following an oscillation of 180° (SB_s180_) (Figure 1B). In the Romanian school, the gymnast takes off with the arms vertically pointing downwards following an oscillation of 90° (SB_s90_) (Figure 1C). Only few authors have studied somersaulting and each studied only one mode of these techniques separately; none has compared them in a single study: Medved and Tonkovic (1991), Medved et al. (1995) and Mkaouer et al. (2012) studied the 270°’s arms swing technique; Munkasy et al. (1996), McNitt-Gray et al. (2001), Mathiyakom et al. (2006), and Okubo (2012) focused on the 180° technique; Lacouture et al. (1989), Duboy et al. (1994) and Leboeuf et al. (2012) investigated the 90° technique. A larger number of authors have analyzed various characteristics of efficient execution of the back somersault; however, their studies were focused on the connections prior to a back somersault (such as applying round-off, flicflac, salto tempo) (King and Yeadon, 2006; McNitt-Gray, 2001; Sadowski et al., 2005; Sands, 2011). Moreover, only few studies amongst them have compiled kinematic and kinetic variables simultaneously (Lacouture et al., 1989; McNitt-Gray et al., 2001; Mkaouer et al., 2012).

Backswing skills are decisive to successfully and safely perform acrobatic elements in gymnastics. A gymnast must obtain the required quantity of movement at the end of this phase in order to guarantee a high enough rotational aerial phase during the somersault. Gravity is the only force acting on the gymnast during the flight period of a somersault. The main consequence is that the angular momentum is constant between the take-off and landing (based on the principle of conservation of angular momentum). In accordance with the mechanical laws, the take-off’s characteristics (arm swing, leg impulse and velocity of back displacement) will determine both angular momentum of the gymnast during the flight, trajectory of the centre of mass (COM) and total flight time (McNitt-Gray et al., 1994; Sands, 2011). Generally speaking, the somersault results from the coordinated involvement of body parts that is imposed to generate an optimal solution to constraints occurring during the execution [whether external constraint (such as gravity) or internals (such as the relative orientation of body segments)]. This requires optimal force and velocity that are related to the gymnast’s ability to create sufficient momentum enabling body management during rotations (Bardy and Laurent, 1994; McNitt-Gray, 2001; McNitt-Gray et al., 2006).

The aim of the study was to compare the mechanical effects of the three above mentioned arms swing techniques, used during the backswing phase on the elevation of the centre of mass during a standing back tucked somersault. It aimed to identify which of the three techniques results in a more efficient performance of the skill.

## Material and Methods

### Subjects

Five elite male gymnasts (age 23.17 ± 1.61 yrs; body height 1.65 ± 0.05 m; body mass 56.80 ± 7.66 kg) volunteered to take part in this study. There were two gymnasts representing the Chinese School, two gymnasts from the Russian school and one from the Romanian school. But they had indeed been trained by Russian, Romanian and Chinese coaches throughout their careers as being members of the national squad. The inclusion criteria were: to be ranked at the international level with participation in world cups and/or championships; average training volume around 25 hours per week; healthy without any muscular, neurological or tendinitis injuries; able to perform back somersaults on the spot. Furthermore, all gymnasts were requested to fully master the three techniques as part of the inclusion criteria. After being informed on the procedures, methods, benefits and possible risks involved in the study, each subject reviewed and signed a consent form to participate in the study. The experimental protocol was performed in accordance with the Declaration of Helsinki for human experimentation and was approved by the university of Manouba ethical committee.

### Measurements

**Figure 2.**
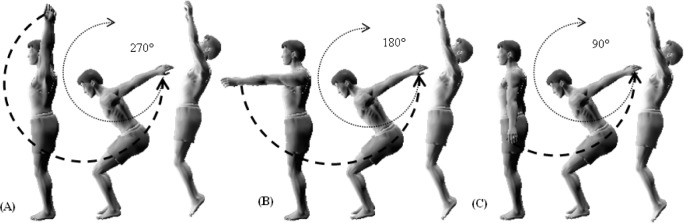
Techniques of arms swing during the preparatory phase of the back somersault. A: 270° arms swing; B: 180° arms swing; C: 90° arms swing.

Gymnasts were requested to perform three different somersaults on three different days. The somersaults differed with regard to the technical performance; in particular the arms’ swing angles in preparation to the take-off. It is worth to notice that all three arms’ swing techniques have two phases: a descending and an ascending phase. The ascending phase is similar in all three schools; it starts when the arms are stretched out behind the back followed by a swing downward and forward to end up in a stretched out position with the arms up. It is the descending phase that actually differs between the techniques. The first position (Figure 2A) applies an oscillation of 270° (SB_s270_). It starts with the arms up, followed by a downward and forward swing up to reaching the backward stretched out position. The second position (Figure 2B) applies an oscillation of 180° (SB_S180_). It starts with the arms stretched out at the front in a horizontal position, followed by a similar swing to the first position and ending in the backward horizontal. Finally, the third position (Figure 2C) applies an oscillation of 90° starting from the anatomical position and ending in the same final shape as the two previous techniques (SB_S90_).

Kinetic data were measured using a Kistler force plate (ref. 9281C, sampling frequency 500 Hz, size 60×40 cm) and analysed using a Bioware Performance Software 5.1.1 (Kistler Instruments, Winterthur, Switzerland). Vertical (F_y_) and horizontal (F_x_) components of force, as well as the centre of mass (COM) velocity (v_x_, v_y_), power (P_x_, P_y_), impulse (I_x_, I_y_) and moments (M_x_, M_y_) were analysed at the moment of the take-off. Vertical (d_y_) and horizontal (d_x_) displacements of the COM during the flight phase were also studied.

For kinematic data, twenty retro-reflective body markers were recorded using two high-speed cameras (250 Hz; HSV-500C^3^, NAC Motion Analysis, Corp., Santa Rosa, CA), in NTSC with VCR C3D and SVHS tape. Body markers were digitized using a video based data analysis system (Movias for Windows 2.0.4). The body segments’ centres of mass were computed using the model of Matshui (1993). Flight time (t_f_), the take-off angle (∠_T_) and the shoulder (∠_S_), hip (∠_H_) and knee joints angles (∠_K_) at the take-off were analysed. Similarly, the angular displacement of these joints (θ_S_, θ_H_ and θ_K_ respectively) and the angular velocity (ω_S_, ω_H_ and ω_K_) were calculated, in the sagittal plane, at the moment of the take-off. The take off angle was calculated using the freeware MB-Ruler version 5.0.

### Procedures

Testing was carried out in the Human Performance Laboratory of the National Centre of Medicine and Science in Sport within a 3-day period, starting at 4:00_PM_ up to 6:00_PM_ under the following environmental conditions: average temperature 23°C. The force plate was synchronized with two high-speed cameras. The first one was placed at the front and the second was sideway at 5m from the centre of the force plate. During all procedures, the participants wore shorts and gymnastic footwear. The warm-up included 10 minutes of light jogging, stretching and several easy jumps with stable landing.

The gymnast started in a standing position on the force plate, with 20 digital markers attached to his body. He was required to randomly “Latin Square (Zar, 1984)” perform one of the standing back tucked somersaults at a precise signal. Three attempts were allowed for each of the somersaults (270°, 180° and 90° arms swing). Each gymnast mastered the three techniques of standing back somersault as part of the inclusion criteria. Plenty of practice had been permitted before the trials under the supervision of the judges. The execution of each somersault was separated by two-minute recovery and five-minute between each technique. Only the best somersault of each technique was registered for the comparative study. Experienced international competition judges marked all trials and helped choosing the best somersaults to be considered for further analysis.

### Statistical Analysis

Data are reported as mean ± standard deviation (SD). Effect size (*d*z) was calculated using GPower^TM^ software [Bonn FRG, Bonn University, Department of Psychology (Faul and Erdfelder, 2004)]. The following scale was used for the interpretation of *d*z: < 0.2, [trivial]; 0.2<0.6, [small]; 0.6<1.2, [moderate]; 1.2<2.0, [large]; and >2.0, [very large] (Scanlan et al., 2012). The normality of distribution estimated by the Kolmogorov-Smirnov test was not acceptable for all variables. Therefore, nonparametric tests were applied: the Friedman Test was used to compare all somersaults’ skills while the Wilcoxon rank-sum test was applied to compare the data pair-wise. The results were considered significantly different when the probability was less than or equal to 0.05 (*p* ≤ 0.05). Statistical analyses were performed using the software package SPSS version 13.0 (SPSS Inc., Chicago, IL, USA).

## Results

Table 1 shows all the descriptive kinetic and kinematic variables. These were compared between the three somersaults’ conditions and presented in Table 2. The Friedman test demonstrated that the three arms’ swing techniques (SB_s270_, SB_s180_ and SB_s90_) had different effect on the standing back somersault. The following paragraphs highlight the main findings:

### Kinetic variables (Table II)

Most of the force and power variables increased during the SB_s90_: the horizontal component of force (F_x_) was considerably increased in condition SB_s90_ with respect to other conditions: [(by 60.04% SB_s90_ vs. SB_s180_ with *p* < 0.05) and (by 67.80% SB_s90_ vs. SB_s270_ with *p* < 0.05)]. The same was observed for the vertical component (F_y_): [(by 19.46% SB_s90_ vs. SB_s180_ with *p* < 0.05), (by 9.02% SB_s90_ vs. SB_s270_ with *p* < 0.05)], the horizontal component of power (P_x_): [(by 45.14% SB_s90_ vs. SB_s180_ with *p* < 0.05), (7.60% SB_s90_ vs. SB_s270_ with *p* = 0.686)] and the vertical component (P_y_): [by 22.20% SB_s90_ vs. SB_s180_ with *p* < 0.05].

The vertical component of impulse (I_y_) was increased by 21.91% in condition SB_s90_ compared to SB_s180_ (*p* < 0.05). Similarly, the horizontal component of impulse (I_x_) was increased in condition SB_s270_ with respect to the two other conditions: [(by 26.75% SB_s270_ vs. SB_s180_ with *p* < 0.05) and (by 32.07% SB_s270_ vs. SB_s90_ with *p* < 0.05)].

The momentum of force (M_x_) was considerably increased in condition SB_s180_ with respect to the other conditions at the horizontal axis: [(by 60.59% SB_s180_ vs. SB_s270_ with *p* < 0.05) and (by 54.17% SB_s180_ vs. SB_s90_ with *p* < 0.05)]. Moreover, the momentum of force’s vertical component (M_y_) was increased in condition SB_s270_ with respect to other conditions: [(by 34.05% SB_s270_ vs. SB_s180_ with *p* < 0.05) and (by 40.87% SB_s270_ vs. SB_s90_ with *p* < 0.05)]. Likewise, the vertical elevation of the COM (dY) was increased: [(by 3.73% SB_s270_ vs. SB_s180_ with *p* < 0.05), (by 6.25% SB_s270_ vs. SB_s90_ with *p* < 0.05)] and similarly for the (COM)’s horizontal velocity (v_x_): [(by 34.23% SB_s270_ vs. SB_s180_ with *p* < 0.05), (by 37.58% SB_s270_ vs. SB_s90_ with *p* < 0.05)].

Interestingly, vertical velocity (v_y_) and the horizontal displacement (d_x_) was no found between conditions.

### Kinematic variables (Table II)

The flight time (t_f_) was increased in condition SB_s270_ and SB_s90_ with respect to SB_s180_ conditions: [(by 8.15% SB_s270_ vs. SB_s180_ with *p* < 0.05), (by 7.28% SB_s90_ vs. SB_s180_ with *p* < 0.05)]. Similarly, the take-off angle (∠_T_) was increased: [(by 2.81% SB_s270_ vs. SB_s180_ with *p* < 0.05), (by 3.60% SB_s90_ vs. SB_s180_ with *p* < 0.05)]. The same was observed for the angle of shoulder joint that was increased at the take-off (∠_S_) by 18.14% during SB_s90_ compared to SB_s180_ (*p* < 0.05).

The angular displacement of the knee joint (θ_K_) was increased in condition SB_s270_ with respect to the other conditions: [(by 17.87% SB_s270_ vs. SB_s180_ with *p* < 0.05) and (by 25.35% SB_s270_ vs. SB_s90_ with *p* < 0.05)].

Angular displacement of the shoulder joint (θ_S_) and hip joint (θ_H_) did not vary during the different conditions. In the same way, the hip joint’s angle (∠_H_) and knee (∠_K_) remained almost identical at the take-off. Also, the angular velocity of shoulder joint (ω_S_), hip joint (ω_H_) and knee joint (ω_K_) were approximately equal (Table 1).

In order to increase reliability of the outcomes, we used ‘the overall effect size and the size of the effect’ at each of the trial in order to compensate for the sample size. The results showed a moderate magnitude to very large in statistical power analyses (Table 2).

Table 3 provides a softer overview of the main variables’ variation between the three conditions mentioned above.

## Discussion

Taking into consideration the small sample size, we analysed the overall effect size and the size of the effect. As shown in Table 2, there was a moderate to strong magnitude in the power size (dz). Even though our gymnasts had experienced the three different schools during their careers, none could deny the fact that each had more or less a preference to perform one technique. One could argue that this preference could have biased the outcomes of the study, however, having reached a power size average of (3.07±1.71), the above results could be considered reliable. Moreover, all gymnasts were requested to fully master the three techniques as part of the inclusion criteria. Plenty of practice had been permitted before the trials under the supervision of the judges. Furthermore, each gymnast was allowed 3 attempts for each technique and only the best attempt was registered for further analysis. Two crucial criteria are considered when assessing the technical performance of a standing back tucked somersault in gymnastics: vertical elevation of the gymnast’ COM and stable landing on the spot without backward displacement. With a better elevation of the COM, the stability of landing is much more secured particularly when combined with a significant 360 degrees rotation enabling the somersault.

**Figure 3.**
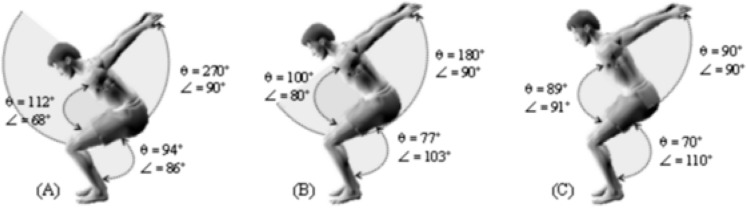
Range of motion variation of knee joint according to techniques of backswing. A: SB_s270_; B: SB_s180_; C: SB_s90_.

One of the main findings of this study is that different techniques of arm swing significantly affected the range of motion (ROM) of the lower limbs during the take-off phase. The angular displacement (θ_z_) of the knee joints during the preparatory phase, prior to a back somersault (bending position), was significantly higher in SB_s270_ than in SB_s180_ and SB_s90_ (*p* < 0.05), and the ROM in SB_s180_ was more important than in SB_s90_ (Figure 3A, B and C). This variation of ROM during the backswing techniques could be explained by the time allocated to the arms swing, which was more important when the degree of oscillation was greater. Salles et al. (2011), Moran and Wallace (2007) and Mathiyakom et al. (2006) have reported the effect of knee ROM on vertical jump. Their findings were in agreement with the present study, where gymnasts produced their peak vertical displacement at an angle of 90° approximately.

Furthermore, if the angular displacement of the arms is larger, the flexion of the hip joint is more important. Clansey and Lees (2010) suggested that there exists a strong relationship between the ROM of the knee and the hip joint during vertical jump. This could explain the large amplitude of the knee flexion during the SB_s270_.

The force generated during the take-off varied significantly, at *p* < 0.05, between the somersaults. The SB_s270_ showed the lowest indices of horizontal force (Fx) followed by SB_s180_ and SB_s90_. The greatest indices of vertical force (Fy) were attained during the SB_s90_ followed by SB_s270_ and SB_s180_. Medved et al. (1995), Medved and Tonkovic (1991) and Lacouture et al. (1989) have reported similar values of the horizontal force developed during the SB_s270_ and vertical force during the SB_s90_, respectively.

The momentum of force varied significantly between the somersaults’ take-offs at *p* < 0.05. The SB_s180_ showed the highest value in the horizontal axis (M_x_) followed by SB_s90_ and SB_s270_. The greatest momentums of force’s vertical component (My) were attained during the SB_s270_ followed by SB_s180_ and SB_s90_. These findings are in accordance with the preceding results of the knee ROM. The best momentum (M_y_) was generated at a knee angle of flexion around 90°, with a technical arms swing of 270°.

Vertical velocity of the COM was comparable at the take-off for all arm swings’ techniques. However, it varied for the horizontal velocity: the SB_s270_ condition displayed significantly higher values (*p* < 0.05) than the SB_s180_ and the SB_s90_ ones.

Vertical and horizontal power developed during the take-off varied significantly between somersaults (*p* < 0.05); the SB_s90_ and the SB_s270_ showed the highest values, while they remained relatively low in the SB_s180_ condition. This drop of the power in the intermediate position of arms swing “SB_s180_” can be attributed to the knees and hips’ ROM, which appeared to be inadequate to produce an important force torque.

At the take-off, the horizontal component of the impulse varied considerably (*p* < 0.05); the SB_s270_ showed the highest values, while the SB_s180_ and SB_s90_ remained relatively similar. Also, on the vertical axis, the impulse changed significantly at *p* < 0.05, whereas the SB_s180_ showed the lowest values followed by SB_s270_ and SB_s90_. As it has been observed earlier for the power, the ROM achieved during SB_s180_ did not lead to an important impulse.

**Figure 4.**
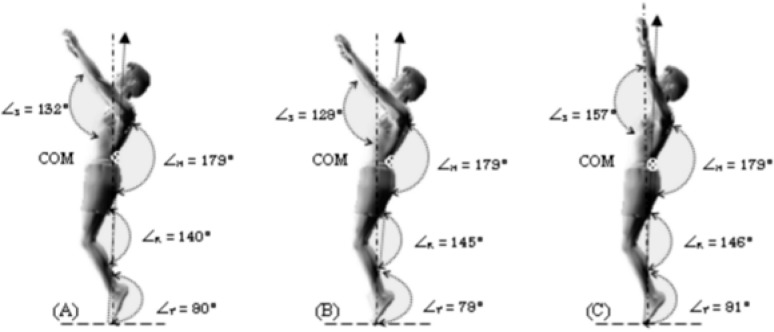
Take off and segmental angles of the three techniques of backswing. A: SB_s270_; B: SB_s180_; C: SB_s90_.

When gymnasts left the floor, their body position varied significantly (*p* < 0.05) depending on the technique used. The take-off angle in the SB_s180_ condition seemed to be relatively more inclined to the vertical line than in the SB_s270_ and SB_s90_ conditions (Figure 4A, B and C). Duboy et al. (1994) and Lacouture et al. (1989) have reported that the optimal take-off angle is around 86° in their study of the kinematic and kinetic characteristics of the standing back tuck somersault. According to Sadowski et al. (2005), during back somersaults the take-off angle of every gymnast was characterized by a different position on the x, y and z axis, however all of them are within a small restricted range of 7° before and 5° after the vertical line.

The joint angles were almost identical during the take-off for all technical backswings, except for the shoulder joint where it significantly varied (*p* < 0.05). During SB_s90_, the angle of arm/trunk was mostly open, while the other two techniques blocked the arms’ action at an angle around 130° (Figure 4).

When the gymnasts were leaving the floor, we noted that the arms’ swing techniques affected the flight phase. The flight time varied significantly (*p* < 0.05): it was longer during the SB_s270_ than during the SB_s90_ and the SB_s180_. The flight times in SB_s270_ and SB_s90_ were comparable to the data published by Mkaouer et al. (2012), Medved et al. (1995) and Medved and Tonkovíc (1991). Also, the vertical elevation of the COM varied considerably in the flight phase (*p* < 0.05). The maximum peak was attained in SB_s270_ followed by SB_s90_ and SB_s180_.

Gymnasts changed their take-off strategy depending on the arms’ swing techniques. In the preparatory phase, prior to a back somersault, we observed that the flexion of knee and hip joints is larger in the SB_s270_ than in the two other conditions. This flexion allowed better vertical elevation of COM, greater momentum in vertical axis, more flight time and a minimum loss of horizontal force. Moreover, it seemed to favour a higher speed and a larger impulse on the horizontal axis. On the other side, we observed the minimum values of all indices of force, power and impulse on the vertical axis during the SB_s180_ condition. In this intermediate position of arm swing and knees flexion, gymnasts performed the back somersault with a minimum loss of energy, but a medium performance in the vertical displacement.

Ultimately, considerable force, power and impulse were observed by the lower limbs during the SB_s90_. This could be explained by the fact that gymnasts were trying to compensate for the small arm swings of the condition by developing extra forces. This technique seemed to be more explosive.

## Conclusion

The aim of the study was to compare the mechanical effects of three arms’ swing techniques (SB_s270_, SB_s180_ and SB_s90_) used during the backswing phase in the completion of a standing back tucked somersault. It ultimately aimed to identify the technique that resulted in a more efficient performance of the skill. Gymnasts changed their take-off strategy according to the arms’ oscillation. In the preparatory phase, the SB_s270_ condition presented a flexion of the knees as well as an inclination of the trunk that were more important than the other two conditions. This range of motion seemed to allow for an important vertical elevation of the COM, a better momentum in the vertical axis and a longer flight time. In addition, it allowed a minimum loss of power on the horizontal axis. Subsequently, the SB_s270_ seemed to favour both high velocity and impulse on the vertical axis. For the SB_s180_ condition, this intermediate arms’ oscillating position seemed to favour the performance of a standing back tuck somersault with a minimum of energy. As for the SB_s90_ condition, greater values of strength, impulse and of power on both the vertical and horizontal axis were observed. This condition seemed to be more explosive and induced more loss of energy in the horizontal axis. In conclusion, considering that the higher elevation of centre of mass in the flight phase would allow best performance and lower the risk of falls, particularly when combined to a great angular momentum, this study demonstrated that optimal arms’ swing technique prior to back tucked somersault was 270°.

## Figures and Tables

**Table 1:** Descriptive statistics of the three techniques of arm swing.

Variables		Descriptive Statistics		
		SB_s270_ (x̄ ± σ)	SB_s180_ (x̄ ± σ)	SB_s90_ (x̄ ± σ)
Kinematic	t_F_ (s)	0.63 ± 0.02	0.58 ± 0.01	0.63 ± 0.01
	∠_T_ (°)	80.74 ± 0.95	78.47 ± 0.43	81.40 ± 0.52
	∠_S_ (°)	132.75 ± 6.52	128.81 ± 7.63	162.16 ± 6.77
	∠_H_ (°)	179.25 ± 0.68	179.51 ± 0.21	179.24 ± 0.40
	∠_K_ (°)	139.06 ± 6.70	145.37 ± 4.85	145.96 ± 4.62
	θ_S_ (°)	237.08 ± 5.04	220.39 ± 3.88	243.93 ± 6.58
	θ_H_ (°)	124.55 ± 7.00	140.98 ± 7.79	146.67 ± 9.35
	θ_K_ (°)	94.13 ± 7.89	77.31 ± 5.56	70.27 ± 1.56
	ω_S_ (°/s)	1126.50 ± 113.84	1169.10 ± 145.11	1135.21 ± 149.52
	ω_H_ (°/s)	767.60 ± 130.52	720.20 ± 151.40	742.76 ± 173.8
	ω_K_ (°/s)	795.7 0 ± 104.61	775.10 ± 106.43	713.39 ± 89.56

Kinetic	F_x_ (N)	124.59 ± 13.56	154.59 ± 8.88	386.91 ± 110.31
	F_y_ (N)	1663.89 ± 68.30	1473.05 ± 69.41	1828.92 ± 91.17
	M_x_ (Nm)	17.86 ± 8.07	45.32 ± 4.06	20.77 ± 6.97
	M_y_ (Nm)	14.95 ± 2.55	9.86 ± 0.96	8.84 ± 0.74
	I_x_ (N·s)	8.45 ± 0.80	6.19 ± 2.01	5.74 ± 1.70
	I_y_ (N·s)	188.33 ± 10.97	149.33 ± 17.40	191.23 ± 5.28
	P_x_ (W)	19.22 ± 4.37	11.41 ± 1.11	20.80 ± 2.52
	P_y_ (W)	4014.21 ± 628.86	3131.93 ± 465.30	4025.86 ± 113.95
	d_x_ (m)	0.022 ± 0.01	0.029 ± 0.01	0.04 ± 0.02
	d_y_ (m)	0.51 ± 0.05	0.44 ± 0.03	0.48 ± 0.04
	v_x_ (m·s^−1^)	0.14 ± 0.01	0.09 ± 0.01	0.09 ± 0.01
	v_y_ (m·s^−1^)	2.93 ± 0.04	2.71 ± 0.28	2.99 ± 0.082

− (∠): angle; (θ): angular displacement; (ω): angular velocity; (d): linear displacement; (T): take off; (S): shoulder joint; (H): hip joint; (K): knee joint; (tf): fly time; (X): horizontal component; (Y): vertical component; (F): force; (v): velocity; (I): impulse; (P): power.

**Table 2:** Comparative statistics of the three techniques of arm swing.

Variables		Friedman Test	SBs_180_ vs. SBs_270_		SBs_90_ vs SBs_270_		SBs_90_ vs. SBs_180_	

			Z	*d*z	Z	*d*z	Z	*d*z
Kinematic	t_F_ (s)	7.6^*^	−2.023^*^	2.10	−1.089	0.24	−2.032^*^	7.03
	∠_T_ (°)	7.6^*^	−2.023^*^	2.26	−1.214	0.70	−2.023^*^	4.34
	∠_S_ (°)	7.6^*^	−1.214	0.67	−2.023^*^	3.14	−2.023^*^	3.65
	∠_H_ (°)	1.6	-NS-	---	-NS-	---	-NS-	---
	∠_K_ (°)	4.8	-NS-	---	-NS-	---	-NS-	---
	θ_S_ (°)	5.2	-NS-	---	-NS-	---	-NS-	---
	θ_H_ (°)	5.2	-NS-	---	-NS-	---	-NS-	---
	θ_K_ (°)	10^**^	−2.023^*^	1.93	−2.023^*^	3.55	−2.023^*^	1.53
	ω_S_ (°/s)	5.2	-NS-	---	-NS-	---	-NS-	---
	ω_H_ (°/s)	1.2	-NS-	---	-NS-	---	-NS-	---
	ω_K_ (°/s)	5.2	-NS-	---	-NS-	---	-NS-	----

Kinetic	F_x_ (N)	10^**^	−2.023^*^	4.42	−2.023^*^	2.66	−2.023^*^	2.29
	F_y_ (N)	10^**^	−2.023^*^	4.38	−2.023^*^	3.92	−2.023^*^	8.53
	M_x_ (Nm)	7.6^*^	−2.023^*^	3.92	−0.674	0.28	−2.023^*^	3.54
	M_y_ (Nm)	10^**^	−2.023^*^	2.93	−2.023^*^	2.51	−2.023^*^	1.29
	I_x_ (N·s)	7.4^*^	−2.023^*^	1.06	−2.023^*^	1.83	−0.944	0.21
	I_y_ (N·s)	8.4^*^	−2.023^*^	2.10	−0.674	0.27	−2.023^*^	2.90
	P_x_ (W)	7.6^*^	−2.023^*^	2.12	−0.405	0.41	−2.023^*^	5.32
	P_y_ (W)	8.4^*^	−2.023^*^	1.43	−0.674	0.01	−2.023^*^	1.91
	d_x_ (m)	0.105	-NS-	---	-NS-	---	-NS-	---
	d_y_ (m)	8.4^*^	−2.023^*^	1.60	−2.023^*^	1.34	−1.753	1.22
	v_x_ (m·s^−1^)	7.6^*^	−2.023^*^	2.80	−2.023^*^	3.80	−0.674	0.37
	v_y_ (m·s^−1^)	2.8	-NS-	---	-NS-	---	-NS-	---

(NS) Not Significant;

(*) Significant at p < 0.05;

(**) Significant at p < 0.01; (Z) Wilcoxon Rank-sum Test; (dz) sample size effect: < 0.2, [trivial]; 0.2–0.6, [small]; 0.6–1.2, [moderate]; 1.2–2.0, [large]; and >2.0, [very large].

**Table 3:** Variation of the main kinetic and kinematic variables at three somersaults

Variables		SB_s270_	SB_s180_	SB_s90_
Kinematic	t_F_ (s)	↗	↘	↗
	∠_T_ (°)	↔	↘	↗
	∠_S_ (°)	↔	↘	↗
	θ_K_ (°)	↗	↔	↘

Kinetic	F_x_ (N)	↘	↔	↗
	F_y_ (N)	↔	↘	↗
	M_x_ (N·m)	↘	↗	↔
	M_y_ (N·m)	↗	↔	↘
	I_x_ (N·s)	↗	↔	↘
	I_y_ (N·s)	↔	↘	↗
	P_x_ (W)	↔	↘	↗
	P_y_ (W)	↔	↘	↗
	d_y_ (m)	↗	↘	↔
	v_y_ (m/s)	↔	↘	↗

*(*↗*) indicates an increase;*

*(*↘*) indicates a decrease;*

*(*↔*) indicates medium value.*

## References

[b1-jhk-40-37] Bardy BG, Laurent M (1994). How do somersaulters control their moment of inertia during flight?. Journal of Sport and Exercise Physiology.

[b2-jhk-40-37] Cheng KB, Hubbard M (2008). Role of arms in somersaulting from compliant surfaces: A simulation study of springboard standing dives. Human Movement Science.

[b3-jhk-40-37] Clansey AC, Lees A (2010). Changes in lower limb joint range of motion in countermovement vertical jumping. Proceedings of the 28th International Society of Biomechanics in Sport.

[b4-jhk-40-37] Domire ZJ, Challis JH (2010). An induced energy analysis to determine the mechanism for performance enhancement as a result of arm swing during jumping. Sports Biomechanics.

[b5-jhk-40-37] Duboy J, Junka A, Lacouture P (1994). Human mechanics: Sports and movements analysis in two dimensions.

[b6-jhk-40-37] Faul F, Erdfelder E (2004). GPOWER: A Priori, Post-Hoc, and Compromise Power Analyses for MS-DOS (Computer Program).

[b7-jhk-40-37] Hara M, Shibayama A, Arakawa H, Fukashiro S (2008a). Effect of arms wing direction on forward and backward jump performance. Journal of Biomechanics.

[b8-jhk-40-37] Hara M, Shibayama A, Takeshita D, Hay DC, Fukashiro S (2008b). A comparison of the mechanical effect of arms swing and countermovement on the lower extremities in vertical jumping. Human Movement Science.

[b9-jhk-40-37] Heinen T, Jeraj D, Vinken P, Velentzas K (2012). Rotational preference in gymnastics. Journal of human kinetics.

[b10-jhk-40-37] King MA, Yeadon MR (2006). A comparison of activation timing profiles for single and double layout somersaults. Journal of Biomechanics.

[b11-jhk-40-37] Lacouture P, Junqua A, Duboy J, Durand B (1989). Dynamographic and cinematographic study of backward somersault. Biology of Sport.

[b12-jhk-40-37] Leboeuf F, Seguin P, Lacouture P (2012). Optimal synthesis versus dynamic analysis for an acrobatic aerial movement. Movement and Sport Sciences.

[b13-jhk-40-37] Marina M, Jemni M, Rodríguez FA, Jimenez A (2012). Plyometric jumping performances’ comparison between elite male and female gymnasts and similar age groups. J Strength Cond Res.

[b14-jhk-40-37] Mathiyakom W, McNitt-Gray JL, Wilcox R (2006). Lower extremity control and dynamics during backward angular impulse generation in forward translating tasks. Journal of Biomechanics.

[b15-jhk-40-37] Matsui S (1993). Center of gravity of the human body. MOVIAS for Windows.

[b16-jhk-40-37] McNitt-Gray JL, Munkasy B, Welch M (1994). External reaction forces experienced by gymnasts during the take-off and landing of tumbling skills. Technique.

[b17-jhk-40-37] McNitt-Gray JL, Hester DM, Mathiyakom W, Munkasy BA (2001). Mechanical demand and multi-joint control during landing depend on orientation of the body segments relative to the reaction force. Journal of Biomechanics.

[b18-jhk-40-37] McNitt-Gray JL (2001). Impulse generation during jumping and landing movements In: Proceedings of the Biomechanics Symposia.

[b19-jhk-40-37] McNitt-Gray JL, Requejo PS, Flashner H (2006). Multijoint Control Strategies Transfer Between Tasks. Biological Cybernetics.

[b20-jhk-40-37] Medved V, Tonkovíc S (1991). Method to evaluate the skill level in fast locomotion through myoelectric and kinetic signial analysis. Med and Biol Eng and Comput.

[b21-jhk-40-37] Medved V, Tonkovíc S, Cifrek M (1995). Simple neuro-mechanical measure of the locomotors skill: an example of backward somersault. Medical progress through technology.

[b22-jhk-40-37] Mkaouer B, Jemni M, Amara S, Chaabèn H, Tabka Z (2012). Kinematic and kinetic analysis of counter movement jump versus two different types of standing back somersaults. Science of Gymnastics Journal.

[b23-jhk-40-37] Moran KA, Wallace ES (2007). Eccentric loading and range of knee joint motion effects on performance enhancement in vertical jumping. Human Movement Science.

[b24-jhk-40-37] Munkasy BA, McNitt-Gray JL, Michele D, Welch MD (1996). Kinematics prior to contact in landings preceded by rotation.

[b25-jhk-40-37] Okubo Y, Cutting-Edge Technology and Rehabilitation (2012). Muscle activity during back tuck somersault. Neuroplasticity, Motor control.

[b26-jhk-40-37] Sadowski J, Boloban V, Wisniowski W, Mastalerz A, Niznikowski T (2005). Key components of acrobatic jump. Biology of Sport.

[b27-jhk-40-37] Salles AS, Baltzopoulos V, Rittweger J (2011). Differential effects of countermovement magnitude and volitional effort on vertical jumping. European Journal of Applied Physiology.

[b28-jhk-40-37] Sands WA, Jemni M (2011). Biomechanics for gymnastics. The Science of Gymnastics.

[b29-jhk-40-37] Scanlan AT, Dascombe BJ, Reaburn PRJ (2012). The construct and longitudinal validity of the basketball exercise simulation test. J Strength Cond Res.

[b30-jhk-40-37] Zar JH (1984). The Latin Square Experimental Design-ultiway Factorial Analysis of Variance. Biostatistical Analysis.

